# Remote consultation with people with eating disorders during the COVID-19 pandemic

**DOI:** 10.1590/0034-7167-2022-0197

**Published:** 2022-11-28

**Authors:** Camila Biscacio Falco, Maria Angélica de Almeida Peres, Jose Carlos Appolinario, Livia Lopes Menescal, Izabella de Góes Anderson Maciel Tavares

**Affiliations:** I Universidade Federal do Rio de Janeiro. Rio de Janeiro, Rio de Janeiro, Brazil

**Keywords:** Binge-Eating Disorder, Anorexia Nervosa, Bulimia Nervosa, Remote Consultation, COVID 19, Trastorno por Atracón, Anorexia Nerviosa, Consulta Remota, Bulimia Nerviosa, COVID-19, Transtorno da Compulsão Alimentar, Anorexia Nervosa, Bulimia Nervosa, Consulta Remota, COVID-19

## Abstract

**Objectives:**

to analyze subjective experiences related to adaptation to remote care by users with eating disorders during the COVID-19 pandemic.

**Methods:**

a descriptive study with a qualitative approach conducted with users of an eating disorders outpatient clinic. A semi-structured remote interview was applied using the Google Meet application. The data were submitted to lexical analysis using ALCESTE software and discussed in the light of scientific evidence.

**Results:**

the remote appointment is a positive strategy but not a substitute for the face-to-face modality. The research cited financial savings, closer contact with professionals, and flexibility of service schedules as advantages. It pointed out the difficulty in clinical evaluation concerning weight, vital signs, and poor mastery of technology as limitations.

**Final Considerations:**

the study induces discussion about the systematization of remote care, which, during the COVID-19 pandemic, were responsible for providing a greater sense of support to people with eating disorders.

## RESUMO

**Objetivos:**

analisar as experiências subjetivas relacionadas à adaptação ao atendimento remoto por usuários com transtornos alimentares durante a pandemia de COVID-19.

**Métodos:**

estudo descritivo de abordagem qualitativa realizado com usuários de um ambulatório especializado em transtornos alimentares. Foi aplicada uma entrevista semiestruturada remota, utilizando-se o aplicativo *Google Meet.* Os dados foram submetidos à análise lexical por meio do *software* ALCESTE e discutidos à luz das evidências científicas.

**Resultados:**

a consulta remota é uma estratégia positiva, mas não substitutiva da modalidade presencial. Vantagens como economia financeira, maior contato com os profissionais e flexibilização de horários de atendimentos foram citadas. Dificuldade de avaliação clínica em relação ao peso, sinais vitais e pouco domínio da tecnologia foram apontados como limitações.

**Considerações Finais:**

o estudo induz a discussão sobre sistematização dos atendimentos remotos, os quais, durante a pandemia de COVID-19, foram responsáveis por fornecer maior sensação de suporte às pessoas com transtornos alimentares.

## RESUMEN

**Objetivos:**

analizar experiencias subjetivas relacionadas a la adaptación a la atención remota por usuarios con trastornos alimenticios durante la pandemia de COVID-19.

**Métodos:**

estudio descriptivo de abordaje cualitativo realizado con usuarios de un ambulatorio especializado en trastornos alimenticios. Fue aplicada una entrevista semiestructurada remota, utilizándose el aplicativo *Google Meet.* Los datos fueron sometidos al análisis lexical mediante *software* ALCESTE *y* discutidos a la luz de las evidencias científicas.

**Resultados:**

la consulta remota es una estrategia positiva, pero no substitutiva de la presencial. Ventajas como economía financiera, mayor contacto con los profesionales y flexibilización de horarios de atenciones fueron citadas. Dificultad de evaluación clínica en relación al peso, signos vitales y poco dominio de tecnología fueron apuntados como limitaciones.

**Consideraciones Finales:**

el estudio induce la discusión sobre sistematización de atenciones remotas, las cuales, durante la pandemia de COVID-19, fueron responsables por fornecer mayor sensación de soporte a personas con trastornos alimenticios.

## INTRODUCTION

On March 11, 2020, World Health Organization (WHO) declared COVID-19 as a pandemic after cases that initially emerged in Wuhan province, China, spread rapidly around the world, causing thousands of deaths in a short period. The virus was named SARS-CoV-2, being considered a betacoronavirus causing the disease entitled COVID 19. The clinical manifestations mainly involve respiratory and infectious symptoms, such as cough, fever, fatigue, anosmia, and dyspnea, among others^(1)^.

Due to the high transmissibility through mucosal contact with contaminated droplets and aerosols, added to the fact that there are, to date, no curative medications for this purpose, government agencies around the world recommended social distancing, use of masks, and hygiene measures. However, while these preventive actions help to reduce the spread of the virus, they also reduce access to resources of the psychosocial protection network, such as leisure, work, contact with family members, and school, among others^(2)^.

Studies have shown that people with eating disorders (ED) have reported worsening symptoms during the pandemic, as well as feelings of loss of support and connection with the health professionals who assist them. Due to the socioeconomic developments arose from the pandemic, many patients with ED have possibly lost some of their influence on the choice of meals and access to the social network due to social distancing measures. Thus, they also began to conduct food inventory practices because of the implementation of lockdown, which can predispose such people to binge eating episodes^(3)^.

Because of the health emergency declared around the world, many health services have reformulated their strategies for aiding, such as adherence to remote appointments to avoid agglomeration of users in the units and, therefore, reduce the spread of the virus^(4)^. In Brazil, professional councils in the health area have expressed themselves regarding the standardization of this assistance model to combat the pandemic, such as the Federal Nursing Council through Resolution COFEN nº 634/2020 and Law nº 13,989/2020, which refers to the use of telemedicine during the health crisis^(5-6)^.

As a result of this event of a health emergency, remote mental health care strategies were being implemented in some services and evaluated in research protocols^(7)^. Such strategies were intensified and disseminated in the pandemic to assist the clientele in a non-face-to-face manner at this atypical moment and have obtained diverse results concerning the assistance to these people^(8)^.

A study conducted in the United Kingdom showed that users with ED felt that the virtual environment was less secure due to the lack of a confidential setting to express themselves freely^(3)^. Another study, conducted in Israel during the COVID-19 pandemic, in turn, showed that the change from the face-to-face to the remote scenario was understood by this clientele as an atypical and necessary condition, far beyond their opportunities to choose. However, under normal conditions, they would not opt for remote care^(9)^.

The treatment of people with ED should be multidisciplinary through a team integrating psychiatrists, clinicians, nutritionists, psychologists, nurses, and other health professionals^(10)^. This team must agree on therapeutic goals, considering the individualities of each case, and its potentialities and difficulties^(11-12)^. Concerning nurses, for example, the field of action is vast. Some objectives of the assistance are aimed at avoiding clinical complications, restoring the eating pattern and weight, stimulating self-control, and strengthening self-esteem^(13)^.

Given the scarcity of studies addressing adaptation to the sudden change in care modality in such a vulnerable clientele, which usually already has difficulties in adhering to treatment, this study was conducted in the Brazilian scenario to subsidize future strategies for the care of people with ED, considering the performance of the multidisciplinary team.

## OBJECTIVES

To analyze the subjective experiences related to adaptation to remote care by users with eating disorders during the COVID-19 pandemic.

## METHODS

### Ethical aspects

The research project, which was created for a master’s thesis, was approved by the Research Ethics Committee following the resolution of the National Health Council n° 466/12^(14)^. The data collection took place after the consent of the participants using a digital signature of the free and informed consent term (TCLE) through the Google Forms platform. The anonymity was guaranteed by utilizing codes in the text, and the confidentiality of the data was ensured with the storage of data in a researcher’s flash drive, avoiding leakage to institutional computers.

### Type of study

It is a descriptive-exploratory study with a qualitative approach, indicated to verify the intensity of the phenomenon studied, articulating theory, praxis, and science in nursing^(15)^. For its development, the study followed the COREQ (Consolidated criteria for reporting qualitative research) checklist criteria)^(16)^.

### Methodological procedures

Researchers conducted a semi-structured interview, whose instrument contained questions about eating habits and routine during the pandemic, as well as an investigation of professional support and clinical picture related to mental health.

With the mediation of the service’s psychiatric fellows, who presented the invitation to the users, the nurse researcher contacted those who agreed to participate to schedule the interviews. These took place on the virtual platform Google Meet, with audio and video recording. Each interview lasted, on average, 40 minutes.

### Study setting

This study was conducted in an eating disorders outpatient clinic, belonging to a university institution in the city of Rio de Janeiro.

### Data source

The study included 16 users diagnosed with anorexia nervosa, bulimia nervosa, or binge-eating disorder and their subclinical forms (DSM-5)^(17)^. It included participants between 18 and 60 years of age, who had been linked to the service for at least six months until the data collection period. The survey excluded participants with less than 50% adherence to face-to-face and/or remote multidisciplinary consultations during the COVID-19 pandemic.

### Collection and organization of data

Collection of data occurred between March 01 and April 29, 2021, and 16 female participants were interviewed until data saturation was verified. The interviews were transcribed in their entirety for data analysis.

After transcription, a unique corpus was prepared and then processed by the computer program *Analyse Lexicale par Context d’un Ensemble de Segments de Texte* (ALCESTE) - version 2010, in the default parameterization. The software performed a lexical analysis of the text and defined the word classes according to their occurrence and co-occurrence, aggregating those that had greater associative strength among themselves and chi-square correlation.

### Data analysis

ALCESTE reduced the words of the corpus to their roots and originated 768 analyzable words. Then, it analyzed the vocabulary and proceeded with the division and classification of the text, obtaining a final use of 85%. These procedures resulted in four stable lexical classes: Class 1: professional mental health support received by users with eating disorders during the pandemic; Class 2: socioeconomic impacts of users with eating disorders during the pandemic; Class 3: pandemic developments in self-esteem and self-perception of users with eating disorders; and Class 4: impacts on the eating routine of people with eating disorders during the pandemic. In this article, Class 1 will be analyzed based on the theoretical framework constituted by scientific articles on the topics analyzed.

## RESULTS

The study considered the variables on income, diagnosis of ED and its subtypes, and the time of diagnosis to contextualize the reality of the participants. Thus, all participants identified themselves as female, 43.75% were white, 50% had an age range of 18 to 30 years, 37.5% had incomplete higher education, 37.5% completed graduate school, 75% lived with their families, 43.75% had a family income of one to three minimum wages, and 62.5% were working at the time of data collection. Regarding clinical data, 65.5% of participants had been diagnosed with binge eating disorder one to three years ago.

The research highlights shorthand words related to professional categories commonly found in mental health services, such as “psycolog” (Phi 0.33) and “psychiatr” (Phi 0.33). The word “nutrition” (Phi 0.26) also refers to a professional category specifically present in this service due to the clientele involved. In addition, concerning mental health care, there is the reduced form “convers” (Phi 0.21), which refers to the reception provided in the service, and “profession” (Phi 0.23), about the professionals of the service in a generalized way. Associated with attendance, other reduced words of important frequency are “remote assistance” (Phi 0.22), “assistance_in person” (Phi 0.19), “time” (Phi 0.18), “appointment” (Phi 0.17), “attend” (Phi 0.17), and “assist” (Phi 0.17).

### Remote care as a protective fator

Participants considered remote care a protective factor in the pandemic period. Access to professionals, even remotely, was important to verbalize feelings and be welcomed, as shown in the following reports:

*This follow-up of the service during this whole time with the nutritionist, with the psychiatrist, and especially with the psychologist, I think it was my time to de-stress during the week, talking and speaking everything out.* (Participant 09, UCE 513)

*I was taken in for my pre-existing health problems, I was taken in for my emotional problems by the psychology and psychiatry team, and for my eating disorders by Psychiatry, Psychology, and the nutritionist.* (Participant 05, ECU 259)

**Table 1:** Main words found in the class dictionary

Reduced form	Words and frequency in the corpus	Phi^1^
psycholog	psychologist (female) (49) psychologists(1) psychology(5) psychological(2) psychologist (male)(6)	0.33
psychiatr	psychiatrist(64) psychiatrists(1) psychiatry(7) psychiatric(1)	0.33
nutrition	nutritional(3) nutritionist(34) nutritionists(2)	0.26
th	he thinks (2) they think(1) he thought(3) they thought(1) I thought(13) I think(121)	0.25
serv	service(78) serving(1)	0.23
profession	professionals(18) professional(17)	0.23
remote_assistance	remote_assistance(42)	0.22
convers	conversation(5) conversed(1) talking(6) to talk(13) conversations(1)	0.21
Assistance_in person	assistance_in person(25)	0.19
time	time schedule(15) times schedules(8)	0.18
chang	exchange(13) change(2) changed(1) thousands(1) changed(1)	0.17
attend	attendance(12) attendances(4)	0.17
appointment	appointment(16) appoitments(7)	0.17
assist	assists(5) assist(5) they assisted(1) he assisted(4) it assisted(2)	0.17
they	they (49)	0.15
cont	counting(1) count(1) contact(28) they counted(1) I count(1)	0.15
in person	in-person(8) personally(6)	0.15

1 Correlation coefficient.

### Adaptation to remote care

All participants considered remote care necessary and important support during the pandemic. For some, the transition from face-to-face to remote model did not generate negative modifications compared to the assistance previously offered:

*Being virtual doesn’t impact anything negatively. On the contrary, you feel welcomed, supported, and assisted. I lived it: the psychiatrist and psychologist gave me all the support at all times I needed. In the moments of loneliness, anguish, all the difficult moments that I went through, and they were extremely efficient.* (Participant 11, UCE No. 655)

For some participants, remote care generated an improvement in the quality of care due to the flexibility of schedules to access the service:

*I could schedule a nutritionist very rarely because I could not schedule every 15 days. The schedules were terrible, they didn’t match, the same with the psychiatrist. So, in fact, I managed to do for the first time. Since I entered the service, the whole follow-up is right, multidisciplinary.* (Participant 09, UCE No. 524)

On the other hand, some participants reported a preference for face-to-face care, or mentioned technological limitations that put the quality of care through remote care at risk:

*The limit itself is the limit of technology, which I think, for mental health care, face-to-face care is part of the treatment. This face-to-face care is very much needed, but it is something that is out of their possibility to solve because it is a risky issue for everyone.* (Participant 04, ECU 177)

*It would be a very big problem because every time I go there they weigh me from my back* [...] *they weigh me, they regulate everything. That part of regulating I think would be difficult.* (Participant 14, ECU 796)

The following report also shows the difficulty in accessing psychotropic drugs through virtual prescriptions:

[...] *smaller pharmacies don’t allow it. It made it more difficult to access the drug for these controlled prescriptions.* (Participant 04, UCE 176)

However, despite the limitations, the study reveals that, from the participants’ point of view, remote care was the only viable alternative given the pandemic context, even if there was a preference for face-to-face care.

### Remote care as a future care strategy

Participants also bring suggestions for assistance tools using remote care:

*I don’t know if it is possible in medicine to be by phone, but to call the person, because, in my case, I was not in such a difficult moment of the eating disorder, but, from what I saw in the service, some people were.* (Participant 08, ECU 484)

*Continue with remote calls. They are extremely efficient. And I, who am a person who is extremely resistant to this virtual world, am living proof of this because I have seen how efficient it is when the person serves with capacity, with efficiency.* (Participant 11, ECU 654)

*When I was attended in person, I already had the psychologist’s contact, but I did not have the psychiatrist’s or the nutritionist’s. When you have that more open contact and that option too, I think it makes it a lot easier. I think that’s it, using technology to our advantage.* (Participant 09, UCE 531)

## DISCUSSION

The reformulation of health care due to the pandemic context has raised discussions around the world regarding the adaptation not only the professionals but also of the users in the face of a possible new care model, in this case, the remote^(18)^. It is undeniable that adaptation to remote treatment varies according to the singularities of each subject. While, in this study, some users adapted easily and listed different advantages of this new modality, others felt more afraid to adhere to the new treatment model.

### Remote care as a protective factor

Despite ambivalence in the responses, remote care was considered, in general, a protective factor against the worsening of the clinical picture associated with the symptoms of sadness. The ease of access to service professionals and the support provided by them were considered relevant points for the maintenance of treatment. The transition from face-to-face to remote care meant that users were not left unassisted, which may have contributed to this result. The integral reception, in the biopsychosocial context, was also a contributing factor to a better adaptation to this new modality.

The scientific literature shows that the implementation of remote care for ED has been promising, with efficacy and satisfaction results similar to face-to-face care, which may be advantageous in the pandemic^(19)^. From the users’ point of view, remote care can increase accessibility to mental health care, favor the flexibility of appointments, reduce time and cost with travel, favor adherence and reduce the risk of contagion by COVID-19^(8,20)^. As the service studied serves people from all over the Metropolitan Region of Rio de Janeiro and works in the morning shift, the modality of care in question allowed for some users a higher frequency of consultations, a fact also observed in other studies.

Thus, although some participants preferred face-to-face care, all generally recognized the modality as the only viable alternative for maintaining treatment in the pandemic. The participants also related the alternative as a protective factor, having offered a sense of welcome, even if, in some cases, in a limited way.

### Adaptation to remote care

Commonly, people with ED have difficulty maintaining adherence to treatment, which can generally be associated with the type of disorder involved and its severity, low motivation for recovery, greater impulsivity, and associated psychiatric comorbidities, among others. Considering these variables, the treatment should be conducted in a welcoming environment, with the establishment of a therapeutic bond, empathy, and collaborative posture of the professional^(21-23)^.

Thus, it is essential to maintain continuity of care in a period in which people, especially those with vulnerabilities, can have even greater consequences on mental health. On the one hand, studies conducted during the pandemic with people with ED have shown that remote service approaches reduce feelings of anxiety, and favor cost reduction in displacement, among others^(3,18)^. On the other hand, some users may present ambivalence regarding adherence to remote treatment due to feelings of self-criticism when viewing themselves on a virtual screen, as well as the feeling of constant concern about the impossibility of monitoring their weight^(18,24)^.

In general, remote care is an alternative to maintain continuity of care but has inherent restrictions to the technology itself, such as difficulty monitoring weight, vital signs, and physical examination^(19)^. These limitations can generate feelings of anxiety in users who need this follow-up. Thus, individualized assessment is a primary tool for good monitoring of users, especially the most severe, and may favor the need to adhere to a hybrid care model.

Although the health body presents an initial difficulty in managing the impacts of the pandemic on the different health services, the research observed that professionals were able to reorganize and formulate alternative strategies to maintain continuity of care in this period of adverse situation. Limitations should be addressed individually, directing singular plans and discussing future care strategies.

### Future care strategies

Participants cite strategies that could be used in remote care to improve the assistance that professionals provide to them, such as access to their telephone contact. In one direction, this strategy undoubtedly configures a greater sense of support for users but, in another, the sharing of professionals’ telephone numbers generates controversial discussions in the context of mental health. Technological resources have become relevant tools during the pandemic to reduce access barriers of health professionals to those in need of assistance. Because it was an unexpected event, in-depth planning of actions had not yet been possible, so the possibilities and limitations of these tools should be considered for learning^(25)^.

However, it is crucial to emphasize that this remote care must occur in an individualized manner and may become exclusive in some cases due to the professional difficulty of remotely approaching users with significant cognitive, visual, and auditory deficits, non-collaborative and psychotic users, for example.

Another difficulty in this strategy is the privacy and confidentiality issues of both users and professionals^(21)^. Although the sharing of the professionals’ telephone numbers can be considered advantageous for users with EDs, ensuring a greater possibility of immediate assistance, it can generate, in professionals, increased workload, problems related to privacy and professional responsibility, as well as difficulty in handling situations of self-aggression and heteroaggression^(21)^.

The thorough systematization in face of all the possible tools and practices to be adopted in this new modality of assistance as well as the long-term use or hybrid model must be discussed and studied regarding its effectiveness, according to the specificities of the clientele and biopsychosocial characteristics of individuals.

### Study limitations

The predominance of participants diagnosed with binge-eating disorder may have been a limiting factor in the study since it hindered the assessment of adaptation to remote service care in people with other ED diagnoses.

### Contributions to the field

This paper discusses the adaptation of a care strategy that was widely used in the context of the health crisis, highlighting its potentialities and weaknesses. In addition, it fosters debate on the need for greater systematization of the modality concerning nursing and other areas of health, as well as reflection on hybrid models in health. This discussion contributes to the teaching, research, and care of people with ED in the context of health care networks in Brazil.

## FINAL CONSIDERATIONS

The study induces discussion about the systematization of remote care, which, during the COVID-19 pandemic, were responsible for providing a greater sense of support to people with eating disorders. This study evidenced that remote care can be a protective factor for people with EDs in pandemic scenarios by maintaining a continuity of care and offering a welcoming space. Adaptation to the remote environment includes advantages such as flexible schedules, while the difficulties are related to complications in evaluating the clinical part, especially concerning weight monitoring.

Participants cited strategies such as telephone calls and access to the personal contact of health professionals on the team as tools for remote care. However, the need for immediate implementation of an assistance model capable of filling the gaps of social restrictions generated by the pandemic did not make it possible to discuss an in-depth systematization regarding the use of various remote tools and strategies. Further studies are needed to understand the magnitude of the potential of this tool and its use in the long term or a hybrid model.
